# *De novo* transcriptome based on next-generation sequencing reveals candidate genes with sex-specific expression in *Arapaima gigas* (Schinz, 1822), an ancient Amazonian freshwater fish

**DOI:** 10.1371/journal.pone.0206379

**Published:** 2018-10-29

**Authors:** Luciana Watanabe, Fátima Gomes, João Vianez, Márcio Nunes, Jedson Cardoso, Clayton Lima, Horacio Schneider, Iracilda Sampaio

**Affiliations:** 1 Laboratório de Genética e Biologia Molecular, Instituto de Estudos Costeiros (IECOS), Universidade Federal do Pará, Campus de Bragança, Pará, Brazil; 2 Centro de Inovações Tecnológicas (CIT), Instituto Evandro Chagas (IEC), Ananindeua, Pará, Brazil; Ohio State University, UNITED STATES

## Abstract

**Background:**

The Arapaima (*Arapaima gigas*) is one of the world's largest freshwater bony fish, and is found in the rivers of the Amazon basin. This species is a potential aquaculture resource, although reproductive management in captivity is limited in particular due to the lack of external sexual dimorphism. In this study, using the 454 Roche platform (pyrosequencing) techniques, we evaluated a major portion of the transcriptome of this important Amazonian species.

**Results:**

Four libraries obtained from the liver and skin tissue of juvenile specimens (representing males and females separately) were sequenced, yielding 5,453,919 high-quality reads. The *de novo* transcriptome assembly resulted in 175,792 contigs, with 51,057 significant blast hits. A total of 38,586 transcripts were mapped by Gene Ontology using Blast2GO. We identified 20,219 genes in the total transcriptome (9,551 in the liver and 16,818 in the skin). The gene expression analyses indicated 105 genes in the liver and 204 in the skin with differentiated expression profiles, with 95 being over-expressed in the females and 214 in the males. The log2 Fold Change and heatmap based on Reads Per Kilobase per Million mapped reads (RPKM) revealed that the gene expression in the skin is highly differentiated between male and female arapaima, while the levels of expression in the liver are similar between the sexes.

**Conclusion:**

Transcriptome analysis based on pyrosequencing proved to be a reliable tool for the identification of genes with differentiated expression profiles between male and female arapaima. These results provide useful insights into the molecular pathways of sexual dimorphism in this important Amazonian species, and for comparative analyses with other teleosts.

## Introduction

The arapaima (*Arapaima gigas*) is one of the most iconic Amazonian species, and one of the world’s largest freshwater fishes, reaching a body length of three meters, and a weight of 200 kg [1]. This species is a member of the order Osteoglossiformes, family Arapaimidae, one of the most primitive groups of teleost fishes [2,3]. *Arapaima gigas* is distributed naturally in Brazil, Ecuador, Peru, and Colombia, and has been introduced into Bolivia. This species is found primarily in the calm waters of the floodplain lakes of the Amazon and its tributaries [4,5]. It is an obligatory air breather, a behavior facilitated by its highly vascularized swim bladder [6,7].

In the wild, *A*. *gigas* typically spawns during the rainy season, when river levels rise [4,8]. The reproductive behavior of the species is complex, which begins with the formation of breeding pairs, followed by nest building, piecemeal spawning, and then infant caregiving behavior, which is provided by the male. Male and female arapaima cannot be distinguished by external characteristics, although some researchers have reported that, during the breeding season, the males may present a more accentuated reddish coloration than the female in the region of the head, tail, and dorsal and anal fins [7,9]. However, this characteristic is not considered to be a reliable criterion for the differentiation of the sexes, given its relatively subtle nature, and the fact that it is observed only in adult individuals during the breeding season, when they form pairs.

The arapaima is a traditional economic resource for human communities of the Amazon basin, but as the species has been exploited commercially since the 18th century, *A*. *gigas* has been listed in Appendix II of CITES (Convention on International Trade in Endangered Species of Wild Fauna and Flora). As a fast-growing fish (1-year-old individuals reach about 10 kg), which is robust, and tolerant of low concentrations of dissolved oxygen, the arapaima also has considerable potential for the Brazilian aquaculture industry [7,10], although the development of effective captive management protocols will require more systematic data on the reproductive physiology, metabolism and genome of the species, in particular for the implementation of artificial spawning techniques.

The recent development of next generation sequencing (NGS) techniques has accelerated research genomics and transcriptomics, in particular on species of commercial interest. One of the most informative strategies for the assessment of gene expression is RNA-Seq, which permits the precise measurement of both quantitative and qualitative levels of gene expression [11,12]. The goal of transcriptome analyses is to investigate functional genome elements in cells and tissues, and their temporal expression, which permits the definition of variation in gene expression among the different types of tissue, organs or life stages of the target organism [13]. In model species, for which information is available on the genome, massive sequencing on platforms that provide small reads (35–100 bps) is performed frequently. In non-model species such as the arapaima, however, pyrosequencing using the Roche 454 platform is preferred because it provides longer sequences (200–400 bp) that facilitate the *de novo* assembly [14]. Indeed, many studies of transcriptome in fish have used this platform [15–20].

In the present study, we used the 454 GS FLX Titanium platform (Roche) to pyrosequence the skin and liver tissue of juvenile arapaima of both sexes. The choice of the skin and liver as target tissues for the evaluation of differential gene expression between the sexes was based on a number of specific traits. Firstly, the skin is the main barrier to harmful substances in the environment, playing a key role in the protection of the organism against pathogens, as well as being important in osmoregulation and ionic exchange, which is reflected in its complex cell structure and composition [21]. As mentioned above, the coloration pattern of the skin is also a potential indicator of sexual differences in adult arapaima in the reproductive phase [9].

The liver, in turn, is the largest hematopoietic organ in fish, which participates actively in the metabolism of proteins, lipids and sugars. Sexual dimorphism has been reported in the liver of some fish, given that this organ contributes to spawning by synthetizing the proteins that are stored in the eggs [22]. The liver is also the principal organ responsible for the synthesis of vitellogenin, a protein that permits the differentiation of male and female arapaima when identified in the plasma of the adult [23].

While the arapaima is an ancient species of considerable economic importance in the Amazon region, scientific studies of the species have focused primarily on its ecology [5,24,25], population genetics [26–29], or focused on specific genes [30,31]. Few data are available on the functional genomics of the species. The largest set of the functional genomic data were collected by Prado-Lima and Val [32] using expressed sequence tags (ESTs), which were obtained from the pituitary of juvenile and adult *A*. *gigas* of both sexes, with the principal differences being found between the juveniles and adults, rather than the two sexes. In the context of these findings, the present study aimed to identify the sexually dimorphic genes in juvenile specimens of *A*. *gigas*, using a relatively non-invasive tissue (the skin) to support the development of a non-lethal tool or procedure for the sexing of individuals of this species. The transcriptome of the liver was also conducted, given that this is the principal organ responsible for the synthesis of vitellogenin, a protein known to discriminate male and female *A*. *gigas* [23], in order to contribute to the genomic database available on this species and other aspects of its reproductive biology.

## Materials and methods

### Ethics statement

The samples analyzed in the present study were obtained from fish farms and collected with the permission of the Brazilian federal environmental, being approved by the Chico Mendes Institute for Biodiversity Conservation (ICMBio), through license number 12773–1 issued to Iracilda Sampaio. All procedures with the fish, since the collection and experimental handling, were performed following of the National Control Board of Animal Experimentation (CONCEA) Law n^o^. 11.974, Federal Council of Veterinary Medicine of Brazil (Law n^o^. 5.517/ Resolution CFMV n^o^. 1000/2012) and the International guidelines defined by U.K. Animals (Scientific Procedures) Act, 1986. The specimens were submitted to euthanasia, using an anesthetic immersion with Benzocaine Hydrochloride. All efforts were made to minimize suffering of the experimental fish.

### Sample collection

Skin and liver tissues were collected from 10 juvenile arapaima specimens (five males and five females), with a mean body weight of approximately 10 kg (+/-1.0 kg) and length of 1 m (+/-0.20 m). These specimens were raised at the Andrera commercial fishery station, located in Bonito, in the state of Pará, Brazil. All samples were collected under the same environmental conditions. The fish sampled for the present study were the offspring of breeding pairs (the principal reproductive mode in this species, in both the wild and captivity) obtained from a semi-intensive farming operation, and represent the F1 generation. The sex and reproductive status of each individual were confirmed through the macroscopic analysis of the gonads. All the specimens were at gonadal development stage I, corresponding to the immature stage in the classification proposed by Lopes and Queiroz [33]. Immediately after tissue collection, the samples were stored in 1 mL of RNA (Ambion, Life Technologies, USA) at 4°C for 24 hours, and then transferred to an ultrafreezer at -80°C.

### RNA extraction and purification

The total ribonucleic acid (RNA) was isolated from the skin and liver tissue of the male and female specimens using a PureLink® RNA mini kit (Ambion, Life Technologies, USA) according to the manufacturer’s instructions. Equal concentrations and volumes of the tissue sampled from different individuals were combined to produce four RNA pools, separated by sex and type of tissue. The samples were treated with RNAse-free DNAse (Invitrogen, CA, USA) to remove any DNA contaminants. The amount and quality of the extracted RNA was determined by using a PicoDrop spechtophotometer (Picodrop, United Kingdom) and a 2100 Bioanalyzer (Agilent Technologies, Santa Clara, CA, USA). All RNA samples were then treated with RiboMinus (Invitrogen, CA, USA), according to the manufacturer’s instructions, to remove the ribosomal RNA (rRNA). The RNA samples were then stored at –80ºC prior to the pyrosequencing reaction.

### Construction of the cDNA library

Four *A*. *gigas* libraries were established: Male liver, Female liver, Male skin, and Female skin. Complementary DNA (cDNA) libraries were constricted in two steps, firstly, the fragmentation of the total RNA and then the synthesis of the double-stranded cDNA. The initial amount of RNA used in the fragmentation was approximately 600 ng/μ, with zinc chloride (ZnCl_2_) and Tris-HCL being added to initiate the fragmentation process. The RNA samples were then analyzed in a 2100 bioanalyzer (Agilent Technologies, Santa Clara, CA, USA), using the RNA 6000 Pico kit. The double strands of the cDNA were then obtained using the cDNA Synthesis System kit (Roche), with the following four steps: denaturation of the RNA by the addition of randomic primer, synthesis of the first cDNA strand, synthesis of the second cDNA strand, and purification of the double cDNA strands. The irregular ends of the cDNA fragments generated by this process were then repaired, after which, adapters were incorporated into both ends of the cDNA strands. Finally, the cDNA fragments were quantified using a TBS 380 QuantiFluor fluorometer (Promega, USA), according to the manufacturer’s instructions. The size the cDNA fragments was verified in the bioanalyzer (Agilent Technologies, Santa Clara, CA, USA) using the High Sensitivity DNA chip. Barcodes were used to identify each library individually.

### EmPCR and 454/GS-Titanium sequencing

The cDNA fragments of *A*. *gigas* were sequenced in the 454 GS FLX Titanium platform (Roche, Branford, CT, USA). The emulsion PCR (emPCR) was based on the enrichment, purification and preparation of the Pico Titer Plate (PTP), which were conducted according to the manufacturer’s instructions. All the libraries were sequenced four times and each run used a PTP with two regions. Raw reads data that support the findings of this study have been deposited in the National Center for Biotechnology Information (NCBI) Sequence Read Archive (SRA) database: (Bioproject: PRJNA353913/**SRX2375194, SRX2375196, SRX2375197, and SRX2375191**).

### Pre-processing reads and *de novo* assembly

The raw data were pre-processed to remove the adapters and low-quality reads (quality score Q>20). The adapters were removed in Geneious v 7.1.7 [34], and the SFF (Standard Flow Format) files were converted into FastQ. The ribosomal RNA reads were removed using SortMeRNA v 2.0 [35], with the default parameters. The subsequent non-rRNA reads were separated by size (>100 bps), quality (Q>20) and homopolymers (<12) in Mothur v 1.35.1 [36]. After filtering, all the reads of *A*. *gigas* generated by pyrosequencing in the 454 Roche platform were assembled using the overlap-layout-consensus algorithm (OLC) [37] in Mira v 4.0.2 [38], which performs well for the de novo strategy. The assemblies were obtained separately for each library, and the results were then clustered in CD-HIT-EST v 4.6.4 [39] to remove redundant sequences, based on a minimum overlap length of 60 bps and a similarity of above 95%. The largest contig was used to represent each cluster in the final assembly.

### Functional annotation

The contigs obtained for the de novo assembly were submitted to BlastX and compared with the RefSeq and Swiss-Prot Protein databases using an E-value cut-off of 1E-5 [40]. The fasta files and their corresponding BlastX outputs were then imported into Blast2GO v. 4.1 [41,42] for functional annotation using Gene Ontology (GO) terminology, based on the evaluation of biological processes (BP), cell components (CC) and molecular function (MF). The functional annotation was also evaluated using the graphic functions and statistics available in Blast2GO v 4.1.

### Analyses of gene expression levels

**Fig 1:**
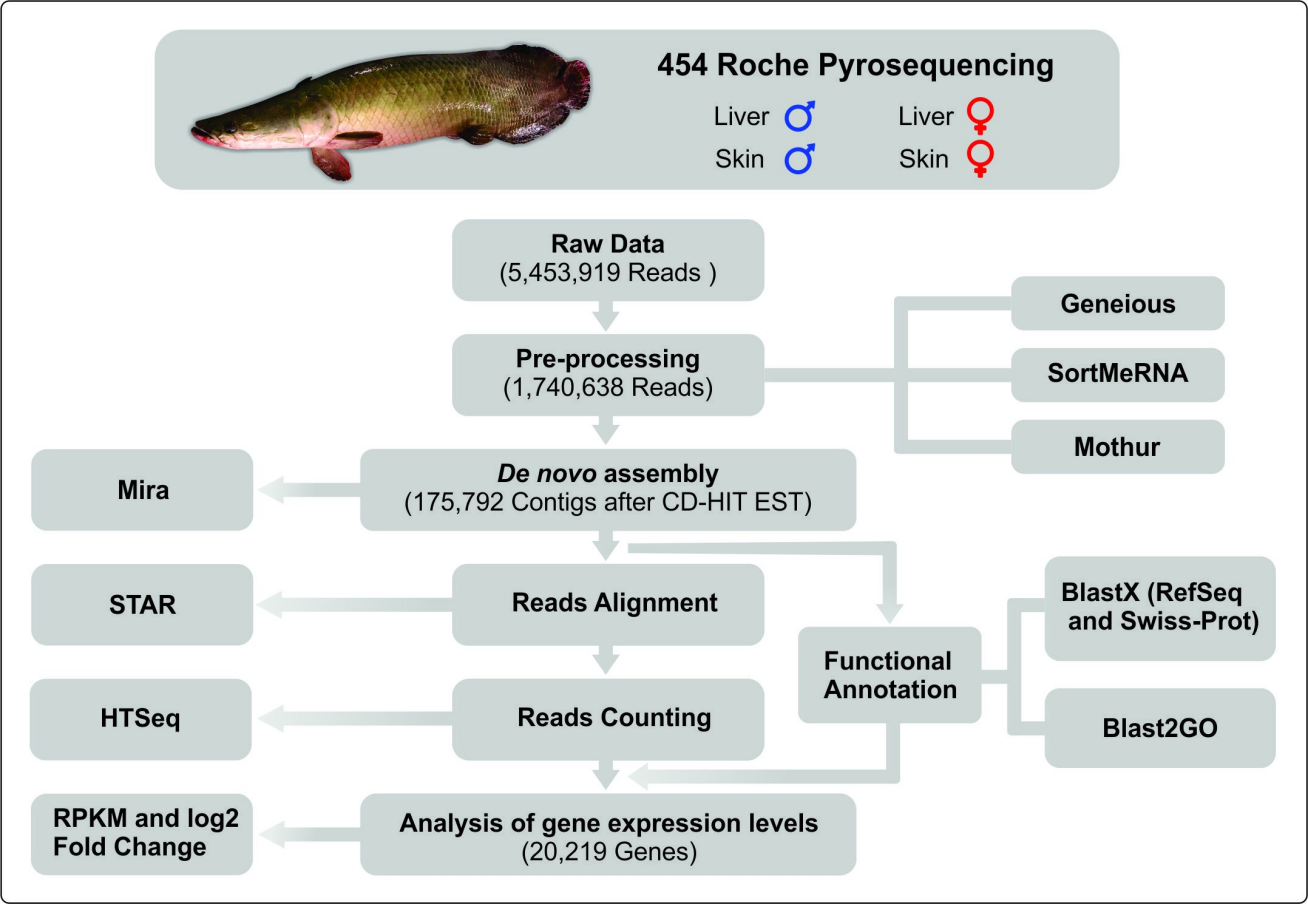
Workflow of the main steps of bioinformatics analysis.

To evaluate the gene expression levels, the raw reads from each library were mapped against the reference transcriptome, based on the entire set of transcripts obtained for *A*. *gigas*, using STAR 2.5 [43]. The counts of the number of aligned fragments in each transcript were obtained using HTSeq 0.6.1 [44]. The RPKM (Reads Per Kilobase per Million mapped reads) was used to normalize the data and calculate the expression of the transcripts in each library using the formula RPKM = (10^9*C)/ (N*L), where C indicates the number of reads mapped for the transcript, N represents the total number of reads in the analysis, and L is the transcript size in base pairs [45]. The expression log2 Fold Change (log2FC) was calculated using the library of the males as the reference for comparisons with the female libraries for both types of tissue. The profiles of the transcripts were considered to be significantly different between males and females when log2FC (Male_RPKM/Female_RPKM) ≥1.0 or log2FC (Male_RPKM/Female_RPKM) ≤-1.0, for genes with RPKM values of at least 100. The heatmap was generated using the Heatmap2 package in the R statistical environment. A Workflow of the main steps of bioinformatics analysis is shown in Fig 1.

## Results

### Sequencing and *de novo* assembly

A total of 5,453,919 raw reads were obtained from the four libraries derived from the samples of the skin and liver tissue of male and female *A*. *gigas* using the Roche 454 platform. After sorting the reads based on their size, sequence quality, the presence of homopolymers, and the removal of ribosomal RNA, 1,740,638 transcripts were identified, with a mean quality of 30.7 and average length of 422.2 bp (Table 1). Even after removing the rRNA sequences using Ribominus, however, a large number of ribosomal reads remained in the sequences, in particular in the liver libraries. It is nevertheless important to note that the remaining rRNA was not a problem for the subsequent transcriptome analyses, given that the rRNA reads were trimmed out in SortMeRNA prior to the assembly of the contigs. In addition, while all the libraries were sequenced in quadruplicate, a smaller number of reads was obtained for the Female skin library, in comparison with all the other libraries, although this difference was normalized for the analysis of gene expression, using the RPKM.

**Table 1:** Summary statistics for the *Arapaima gigas* transcriptome, from the Roche 454 pyrosequencing libraries and the assemblies generated for each library separately.

	Male		Female	
	Skin	Liver	Skin	Liver
**Pyrosequencing reads**				
Raw Reads	1,315,106	1,898,857	580,558	1,659,398
Ribosomal RNA	413,443	1,444,941	162,945	1,119,938
Filtered Reads	750,016	311,348	325,268	354,008
Average Length	393.6	438.6	398.2	458.7
Mean Quality	29.4	32.4	29	32
**Assembly statistics**				
Reads Assembled	362,908	215,813	105,562	243,699
Contigs	108,060	21,597	44,247	25,177
N50 (bps)	742	874	627	920
Largest Contig	8,900	12,525	13,758	7,506
Average Length	710.26	833.12	593.79	876.64
Number of bases	76,751,253	17,994,637	26,273,278	22,075,645

N50: This value was computed by sorting all the contigs from the largest to the smallest, and then determining the minimum set of contigs whose total size is equal to 50% of the entire transcriptome.

The de novo assembly generated a total of 21,597 contigs for the Male liver, 25,177 for the Female liver, 108,060 for the Male skin, and 44,247 for the Female skin (Table 1). The average length of the transcripts and the N50 for each assembly are also shown in Table 1. The largest contig was obtained from the Female skin library, with 13,758 bp. The total number of contigs obtained by the *de novo* assembly of the four libraries was 199,081, including all those transcribed and those that were common to the four libraries. In order to obtain a non-redundant transcriptome, all identical transcripts were removed using CD-HIT-EST, as described in the Methods section. Following the removal of the redundant sequences, a total of 175,792 contigs were reconstructed for the transcriptome of *A*. *gigas*, with sizes ranging from 100 to 13,758. The total number of base pairs was 126,209,063. Mean transcript size was 717.95, and the content of adenine and thymine bases was approximately 56.06%, while guanine and cytosine represented 43.93% of the whole arapaima transcriptome. Overall, 65.9% of the transcripts varied in size between 500 and 1,000 base pairs (S1 Fig).

### Transcriptome annotation

**Fig 2:**
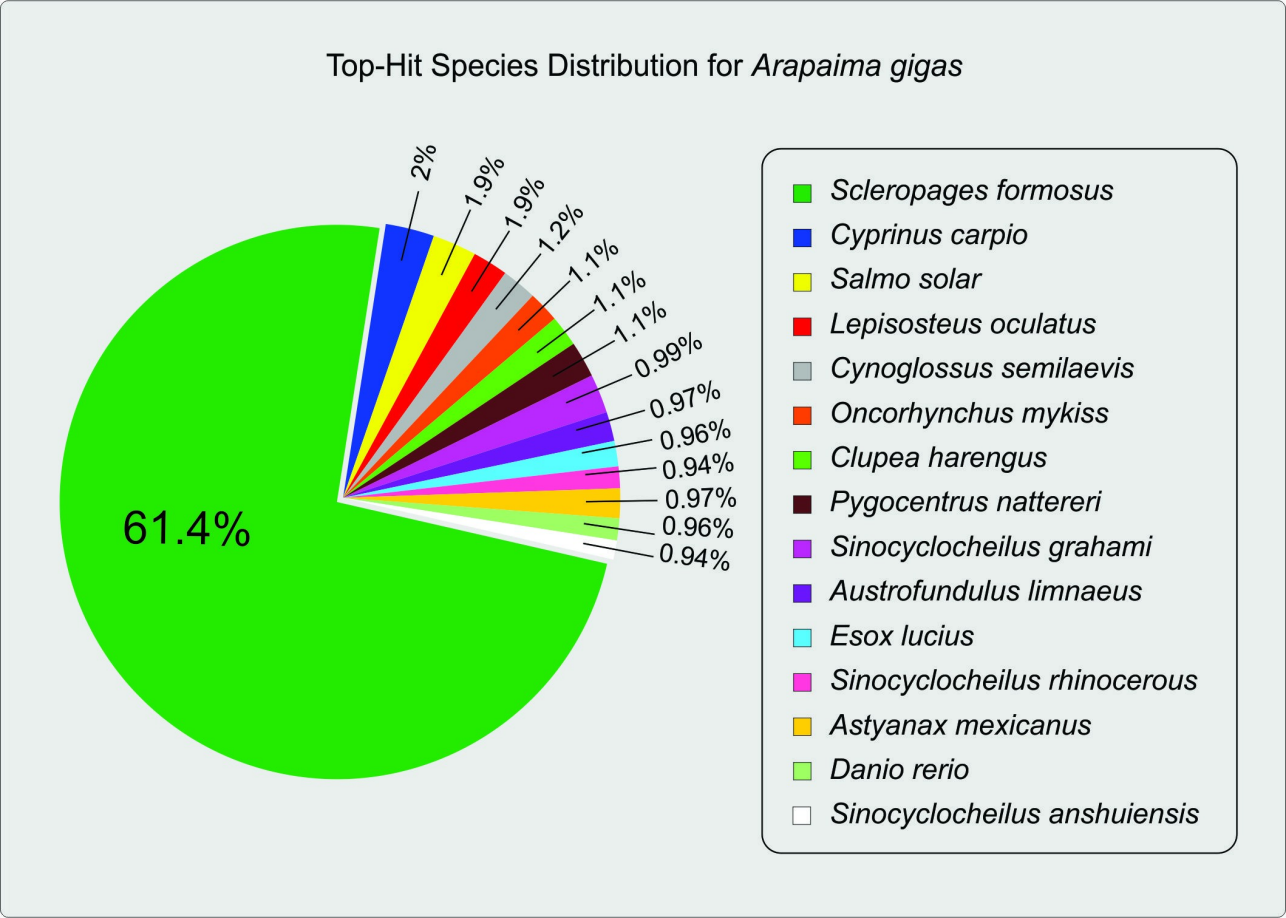
Top-hit species distribution based on BLASTx results.

To interpret the transcriptome of *A*. *gigas*, the 175,792 assembled contigs were compared with the Swiss-Prot and RefSeq datasets after analysis in BlastX, with 51,057 (29.04%) transcripts having significant hits. Of this total, 38,586 contigs (21.9%) were mapped by GO and 34,476 (19.6%) were annotated. The BlastX top-hit species distribution (Fig 2) confirmed the taxonomic affinity of the transcripts with *A*. *gigas*, based on gene and protein homologies. A majority of the transcripts obtained for *A*. *gigas* were also homologous with other fish species, with almost two-thirds (61.4% of the transcripts) being recuperated for the sister species of the arapaima, *Scleropages formosus*.

The transcripts were classified functionally by tissue, sex and the complete dataset, including all the libraries. No marked variation was found among the transcripts from the different datasets when their principal domains were compared. The BP category had the largest number of annotations, representing more than half of the transcripts (~55.6%), followed by MF (~20.5%) and CC (~23.8%). The classification of the complete dataset returned 166,807 annotations for BP, 71,473 annotations for MF and 61,648 annotations for CC.

The distribution of the annotated transcripts in the second level of GO terms revealed a very similar pattern between the males and females, and the complete transcript dataset (S2 Fig). Even so, certain differences were observed among categories in each domain in the comparison between the skin and liver datasets (S3 Fig). In the case of the biological processes (BP), the categories with the largest number of transcripts in all libraries were cellular processes (GO: 0009987), single-organism processes (GO: 0044699), and metabolic processes (GO: 0008152). For cell components (CC), the most numerous categories were cell (GO: 0005623), cell part (GO: 0044464), and organelle (GO: 0043226), while the principal categories observed in molecular function (MF) were binding (GO: 0005488) and catalytic activity, GO: 0003824 (S2 and S3 Figs). Reproduction (GO: 0000003) represented approximately 1.3% of the transcripts of biological processes, on average, in all the datasets.

Despite these similarities in the second-level distribution of GO terms, males and females were slightly different in the “extracellular region” and “extracellular region part” categories of the cell components, with more transcripts in females than in males and the entire transcript dataset. Similarly, the “catalytic activity” category of molecular function was more abundant in the females (S2 Fig). A number of differences were observed in several categories of biological processes when skin and liver tissues were compared, with the largest number of transcripts being recorded in the liver, whereas most categories related to cell components were more abundant in the skin. The principal categories of molecular function were “binding”, “catalytic activity” and “nuclei acid binding transcription factor activity” in the liver and “molecular transducer activity” in the skin (S3 Fig).

### Gene expression levels

A total of 20,219 genes (S1 Table) were identified in the complete transcriptome of the arapaima, with 9,551 genes in the liver and 16,818 genes in the skin, including isoforms. In the liver samples, 2,601 genes were shared between males and females, while 3,066 were active exclusively in the males and 3,884 in the females. In the skin samples, 2,623 genes were shared by both sexes, 11,807 were expressed only in the males, and 2,388 only in the females.

The analyses of gene expression revealed 62 genes that were more active in the male liver of *A*. *gigas* in comparison with the female liver, while 43 genes were over-expressed in the female liver (S2 Table). In the skin, 152 genes were more active in the males, while 52 were over-expressed in the females (S3 Table). The log2FC values were also higher for the genes of the skin library when compared between the sexes, in comparison with those obtained from the liver of *A*. *gigas*.

**Fig 3:**
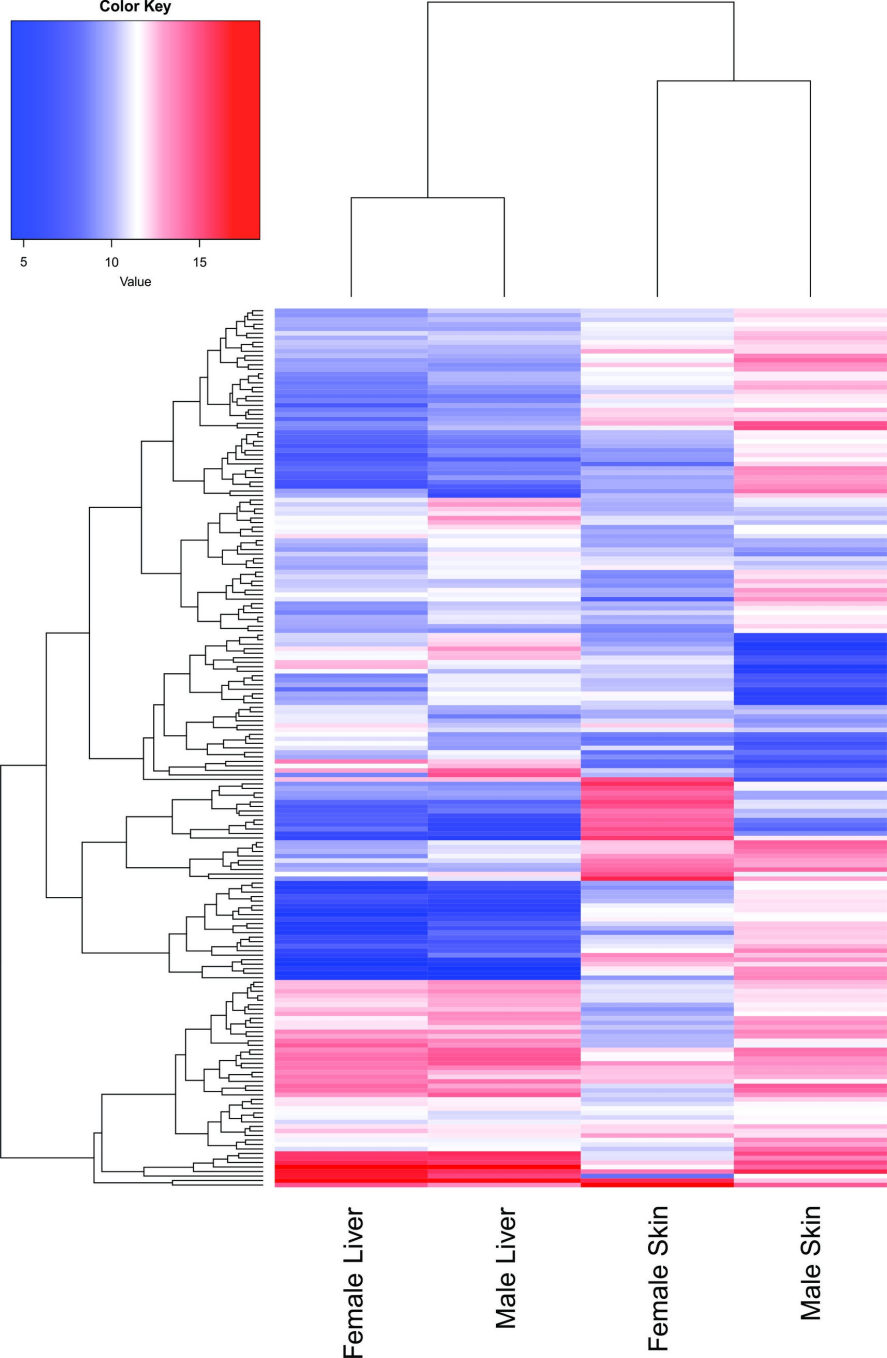
Heatmap of the genes with differentiated expression profile in the liver and skin of males and females of *A*. *gigas*. The colored bars indicate the relative expression levels in RPKM. Highly expressed genes are shown in red, while low expressed genes are shown in blue. The complete list of genes used to generate the heatmap is in S4 Table.

In the heatmap based on the RPKM values (Fig 3), the clustering of the genes from each library indicated similar expression profiles in the liver tissue of the males and females, whereas the clustering of the skin libraries was more differentiated between sexes, with some genes being over-expressed in the males, and others in the females.

The genes most expressed in the liver of the arapaima were Hemopexin (*hpx*), Serotransferrin (*tf1*), Apolipoprotein A-I (*apoc1)* and Fibrinogen- γ (*fbg*) in the males, and Apolipoprotein A-I (*apoc1)*, Hemopexin (*hpx*), Serotransferrin (*tf1*), Fibrinogen- γ (*fbg*) and Fibrinogen-β (*fbb*) in the females (S2 Table). Table 2 shows the genes (highest log2FC values and RPKM ≥100) for each library. In the genes regulating differentially in the liver with the highest log2FC values, Serum amiloyd A-5 (*saa5*) and complex I assembly factor TIMMDC1 (*timmdc1*), mitochondrial (*timmdc1*), up-regulated in the males. On the other hand, the acyl-coenzyme A thioesterase 2 (*acot2*), DNA ligase 1 and SUMO-conjugating enzyme UBC9 (*ubc9*) genes presented the lowest log2FC values, and were up-regulated in the females (Table 2). A number of genes related to reproduction showed differentiated expression profiles in the liver, Ubiquitin (*ubiq*), Calreticulin (*calr*) and Zona pellucida sperm-binding protein 3-like (*zp3*) (S5 Table).

**Table 2:** Genes with a more differentiated expression profile between males and females of *A*. *gigas*.

Gene Symbol^a^	Accession Number^b^	Description^c^	Tissue^d^	Length^e^	RPKM Male^f^	RPKM Female^g^	log2FC^h^
*LOC108934571*	XP_018607998	B-cadherin isoform X1	Skin	3,904	273.744	1.952	7.131
*spaca4*	XP_018609834	Sperm acrosome membrane associated protein	Skin	1,411	472.264	5.401	6.449
*mal*	XP_018606345	Myelin and lymphocyte protein	Skin	1,638	348.480	4.653	6.226
*cola1*	XP_018599340	Collagen alpha-1 (X) chain	Skin	2,718	201.684	2.804	6.168
*ita4*	XP_018584540	Integrin alpha-6	Skin	4,163	358.190	5.492	6.027
*sdc4*	XP_018600166	Syndecan-4 isoform X2	Skin	3,085	154.053	2.470	5.962
*b2m*	NP_001185503	Beta-2-microglobulin precursor	Skin	984	886.748	15.492	5.838
*naca*	XP_005986238	Nascent polypeptide-associated complex subunit alpha isoform X4	Skin	1,485	62.183	10.265	5.775
*tyba*	XP_018592541	Thymosin beta-a	Skin	844	914.665	18.062	5.662
*nadh6*	YP_001816867	NADH dehydrogenase subunit 6 (mitochondrion)	Skin	1778	696.441	14.011	5.635
*arvcf*	XP_018605820	Armadillo repeat protein deleted in velo-cardio-facial syndrome	Skin	724	3.473	178.973	-5.867
*mta1*	XP_018590687	Metastasis-associated protein MTA1 isoform X3	Skin	519	4.845	220.294	-5.506
*glis2*	XP_018600885	Zinc finger protein GLIS2	Skin	765	3.287	129.527	-5.300
*a1m*	XP_018611035	Alpha-1-macroglobulin	Skin	4,784	6.307	183.225	-4.860
*ctnna2*	XP_008509893	Catenin alpha-2	Skin	1,262	7.970	211.391	-4.729
*LOC110446415*	XP_021347235	Uncharacterized protein LOC110446415	Skin	563	4.466	108.308	-4.599
*b3galnt2*	XP_018595532	UDP-GalNAc:beta-1,3-N-acetylgalactosaminyltransferase 2	Skin	484	5.195	110.238	-4.407
*znf384*	XP_015193432	Zinc finger protein 384 isoform X1	Skin	514	4.892	103.804	-4.407
*ret1*	XP_018593664	Retinol-binding protein 1 isoform X1	Skin	940	10.700	186.500	-4.123
*dennd5a*	XP_016120336	DENN domain-containing protein 5A	Skin	696	10.838	164.271	-3.921
*saa5*	XP_018618982	Serum amyloid A-5	Liver	865	1198.344	27.998	5.419
*timmdc1*	XP_018613206	Complex I assembly factor TIMMDC1, mitochondrial	Liver	1,435	103.641	19.690	2.396
*ak2*	XP_018615415	Adenylate kinase 2, mitochondrial isoform X2	Liver	939	148.787	30.091	2.305
*slc6a6*	XP_018602541	Sodium- and chloride-dependent taurine transporter isoform X1	Liver	3,875	394.273	81.250	2.278
*hsp70*	XP_018589419	Heat shock 70 kDa protein	Liver	2,516	128.970	30.482	2.080
*iigp5*	XP_018587675	Interferon-inducible GTPase 5	Liver	1,712	118.461	28.293	2.065
*LOC108927954*	XP_018597164	Bile salt export pump isoform X2	Liver	1,833	186.862	52.851	1.821
*apmap*	XP_018580468	Adipocyte plasma membrane-associated protein	Liver	1,464	132.372	41.357	1.678
*txnip*	XP_018620258	Thioredoxin-interacting protein isoform X2	Liver	2,347	247.711	77.393	1.678
*gstk1*	XP_018591791	Glutathione S-transferase kappa 1	Liver	781	115.411	361.786	1.673
*acot2*	XP_018618234	Acyl-coenzyme A thioesterase 2, mitochondrial	Liver	3,719	35.143	347.319	-3.304
*dnli1*	XP_016400483	DNA ligase 1	Liver	533	169.113	106.024	-2.648
*ubc9*	NP_001187932	SUMO-conjugating enzyme UBC9	Liver	924	24.387	122.318	-2.326
*eif4ebp3*	XP_018611927	Eukaryotic translation initiation factor 4E-binding protein 3	Liver	2,058	37.228	151.025	-2.020
*agt*	XP_018604956	Angiotensinogen	Liver	2,000	137.457	530.800	-1.949
*fasn*	XP_016106557	Fatty acid synthase isoform X1	Liver	2,327	42.608	150.913	-1.824
*gltscr2*	XP_018598262	Glioma tumor suppressor candidate region gene 2 protein	Liver	1,484	30.369	100.640	-1.728
*cox7c*	XP_018614615	Cytochrome c oxidase subunit 7C, mitochondrial	Liver	400	56.335	181.642	-1.688
*calr*	XP_018586503	Calreticulin	Liver	1,717	73.495	230.388	-1.648
*fba*	XP_018611791	Fibrinogen alpha chain	Liver	2,620	78.690	243.471	-1.629

a) Symbol used to represent the gene

b) Access number in RefSeq

c) Gene Description

d) Tissue where the gene was up-regulated in Arapaima

e) Transcript size in bp

f) RPKM value obtained for the transcript in the *A*. *gigas* male

g) RPKM value obtained for the transcript in the *A*. *gigas* female

h) The absolute value of log2FC ≥ 1 or ≤-1 means the magnitude of up or down-regulation for each gene, positive values indicate up-regulated genes expression in male and negative in female.

In the skin of arapaima, the most expressed genes in males were the NADH dehydrogenase subunit 5 (*nadh5*), uncharacterized protein LOC106513546 (*LOC106513546*), Vimentin (*vim*), Thymosin beta-a (*tyba*) and Beta-2-microglobulin precursor (*b2m*). In females, the most abundant transcripts were related to uncharacterized protein LOC106513546, Transposable element tcb1 transposase (*tcb1*), Sodium/potassium/calcium exchanger 2 isoform X3 (*slc24a2*), RNA-directed DNA polymerase from mobile element, Transposon Tf2-1 polyprotein (*tf2-1*) and B-cell receptor CD22 (*cd22*). The transcripts with the most differential regulation between sexes in the skin included B-cadherin isoform X1 (*LOC108934571*), Sperm Acrosome Membrane-associated Protein 4 (*spaca4*), Myelin and lymphocyte protein (*mal*), Collagen alpha-1 (*coaa1*) and Integrin alpha-6 (*ita6*), which returned the highest log2FC values (Table 2 and S3 Table). The Armadillo repeat protein deleted in velo-cardio-facial syndrome homolog, Metastasis-associated protein MTA1 (*mta1*), Zinc finger protein GLIS2 (*glis2 nkl*), alpha-1-macroglobulin (*a1m*) and catenin alpha-2 (*ctnna2*) genes presented the lowest log2FC values, and were thus up-regulated in the females (Table 2).

### Genes associated with pigmentation

A number of genes associated with pigmentation were annotated in the transcriptome of *A*. *gigas*, but with low RPKM values (S5 Table). The main genes identified here included what encode the premelanosome protein *(pmel)*, genes of melanosome transport essential for melanin synthesis (*matp*), transcription factors for the synthesis of melanin (*pax3*, *mtif* and *sox10*), and the alpha-melanocyte-stimulating hormone receptor (*α-mshr*) and melanocortin receptor 4 (*mc4r*). Few genes were associated with the development of xanthophores (*pax3*, *sox 10*, and *cflrs*) and pteridines (*xdh* and *mycbp2*). As expected, most of transcripts related to pigmentation were founding the skin tissue, even though some of these genes were also expressed in the liver (S5 Table).

## Discussion

Next Generation Sequencing (NGS) has been widely employed for the transcriptome analysis of a range of species, applied to a number of different questions, such as sex differentiation, response to stress, and the identification of genes related to growth and metabolism [46–48]. Nonetheless, no NGS transcriptome data were available for *A*. *gigas* prior to the present study, even though the lack of a reliable procedure for the differentiation of the sexes in juveniles and non-breeding adults represents a major obstacle to the formation of a broodstock for raising *A*. *gigas* in captivity [23,49]. In the present study, we used *de novo* assembly to map a large number of transcripts obtained by pyrosequencing using the Roche 454 platform on samples of the skin and liver of juveniles in order to identify genes with differentiated expression profiles in the males and females of this important fishery resource from the Amazon region.

### Functional annotation

The functional annotation of the contigs in BlastX identified a total of 20,219 genes, including isoforms, a relatively large number in comparison with the NGS data obtained by pyrosequencing in the Roche 454 platform in previous studies with other fishes species [19,50]. Most of the transcripts (61.4%) were similar to those identified in *S*. *formosus*, the sister species of the arapaima, one of the other seven extant members of the family Osteoglossidae [51]. The number of genes predicted by our transcriptome was similar to those recorded for the three varieties of *S*. *formosus*, that is, the golden arowana (22,016 genes), the red arowana (21,256 genes), and the green arowana, with 21,524 genes [52,53].

The functional diversity of transcripts assembled in *A*. *gigas* was analyzed using the Gene Ontology (GO) terms, which provide a reliable and structured dataset for functional genomics, widely used to evaluate specific biological domains and to describe the gene products of distinct organisms. The GO identifies three main categories: (i) biological processes, i.e. the essential molecular events for the function of cells, tissues, organs, or organisms, (ii) molecular functions, which are defined by the biochemical properties of the gene products at a molecular level, and (iii) cell components, which indicate the cell part or extracell environment in which the gene products act [54,55].

The GO functional annotation indicated no marked differences in the distribution of GO terms between the sexes or in the complete transcript dataset, and the principal differences were observed between the two types of tissue, with increased biological processes being found in the liver, reflecting its role as a multifunctional organ, responsible for several essential processes in vertebrates [56]. In the skin, the differences in the expression of the transcripts were related more to cell components, which reflect the fact that the skin is subject to continuous modification, as well as acting as the principal barrier to impacts from the surrounding environment [57]. The GO results were broadly compatible with those obtained for other vertebrates, in which biological processes typically represent more than 50% of the functional annotations, followed by cell components and molecular functions. Prado-Lima and Val [32] obtained similar results for the Expressed Sequence Tags (ESTs) obtained from *A*. *gigas* by cloning.

In the second level of GO categories, most transcripts from the biological processes and cell components are related to the basal maintenance of tissues and cells (e.g., “cellular process” and “cell”), reinforcing the significance of these processes to the organism. In the case of molecular function, most transcripts were related to “binding” and “catalytic activity”. The annotation of the distinct functional categories reveals the diversity of transcripts in *A*. *gigas*, as well as the analytical efficiency of the *de novo* assembly and the pyrosequencing in the 454 Roche platform. Similar results have been reported in other fish species, such as *Oncorhynchus mykiss* [15] and *Coilia nasus* [58].

### Genes most expressed in the transcriptome of the liver and skin of *A*. *gigas*

The analyses of the levels of gene expression revealed distinct profiles between the liver and skin of the arapaima reflecting the high degree of specialization for cell function and type in the two organs. The tissue-specific expression of genes is essential to the development of specificity and complexity in multicellular organisms [59]. The results also indicated that the transcripts expressed in the liver were similar between the sexes, whereas a differentiated expression profile was found between males and females in the skin. However, this difference between males and females skins may be a reflection of the low recovery of transcripts due to the discrepancy in initial reads numbers between the male and female libraries for this tissue.

The liver is an important organ that controls a number of vital physiological functions, playing an active role in processes such as digestion, metabolism, the production of plasmatic proteins, and the detoxification of xenobiotics [56]. The majority of the most abundant genes expressed in the liver are related to the synthesis of plasmatic proteins, such as Apolipoprotein, Hemopexin, Serotransferrin, Serum amiloyd, Serum albumin, and the three genes encoding Fibrinogen. The *hpx* gene was expressed most in the liver of male arapaima, and is associated with the cell response to estrogen in the GO terminology, as well as protecting against oxidative damage, by binding with heme groups. In bony fishes, the *hpx* is referred to as the Warm-temperature Acclimation related 65 kDa Protein (*wap65*), which has two differential forms of expression: *wap65-1* is expressed constantly, while *wap65-2* is expressed only in the liver, and at different levels according to acclimation [60].

The *apoc1* gene, which is expressed intensely in the livers of both males and females, participates actively in the transport and metabolism of lipoproteins. Recently, two isoforms were described that have a role in the gastrulation of zebrafish, reflecting the importance of this protein in other processes [61]. Fibrinogen is a glycoprotein codified by three genes (*fga*, *fgb*, and *fgg*), which are synthesized almost exclusively by hepatocytes, and are responsible for coagulation [62]. All the genes involved in the synthesis of fibrinogen were expressed more intensely in the liver of the female arapaima, confirming the unique characteristics of this gene family in terms of its highly coordinated transcription, and the simultaneous increase or decrease in the expression of the three genes according to the positive or negative regulation [63].

The skin of fish, like that of other vertebrates, is a highly complex organ which not only protects the organism from environmental factors, but also plays a major role in communication, sensorial perception, locomotion, respiration, ionic regulation, excretion, and thermal regulation. This diversity of functions may account for the relatively large number of genes identified in the transcriptome of the skin in comparison with the liver. While the liver is more active, biologically, than the skin, the number of active genes identified in its tissue was much smaller than that found in the skin of *A*. *gigas*. One of the factors that may account for this result is the structural composition of the liver, which is mostly made up of hepatocytes (60–80% of the cells), with other cells including those of the endothelium, Kepffer cells, lymphocytes, and bile cells [64]. The skin is much more complex, being composed of a number of layers formed principally by the dermis and epidermis, in addition to its auxiliary organs, which are being constantly differentiated and renovated, due primarily to its role as an external barrier and protection of the organism. The majority of the genes expressed most intensely in the skin are related to its structure and composition, such as the gene families related to the collagen, mucin, desmoplakin, and vimentin. In spite of the high diversity of genes expressed in the skin, transposable elements (*tcb1* and *tf2-1*) are also highly expressed in the skin females and males of arapaima. Transposable elements were recorded in the genomes of all three varieties of *S*. *formosus*, corresponding to 27% of that of the golden arowana, 27% in the red arowana, and 28% in the green arowana [52,53]. It is well documented that transposable elements and related sequences correspond to about 30% to 50% of most genomes in mammals [65] with higher values being reported in amphibians and plants. Therefore, the high expression of these elements reveals that they are transcriptionally active in the skin of arapaima.

### Gene expression in the liver of the male and female *A*. *gigas*

The liver is a multifunctional organ, and the second most complex in vertebrates, and is even sexually dimorphic in some organisms [22,56]. When the liver libraries of the male and female arapaima were compared, the highest log2FC value was recorded for the *saa5* gene, which was over-expressed in the males. Serum amiloyd A (*saa*) is one of the principal genes involved in the immunological response of the organism during the acute inflammatory stage provoked by infection, trauma or stress [66]. The expression of this gene has been recorded in a variety of tissues in a number of different vertebrates, although the liver appears to be the primary organ synthesizing *saa* [67].

The *ubc9* gene, a member of the ubiquitin-conjugating enzyme family, is involved in essential biological processes, such as the regulation of the cell cycle, cell proliferation, and DNA repair [68,69], and was one of the genes with more differentiated expression profile in the liver of female arapaima. In addition to regulatory processes, the *ubc9* protein regulates the development of ovarian follicles [70]. A recent study of the fish *Cynoglossus semilaevis* indicated that this gene is also involved in oogenesis, gametogenesis, and sex reversal by controlling the other genes involved in these processes [71].

After the gonads, the liver is the organ that has the largest number of genes involved in sex-linked expression, although the potential of the expression profile of the liver for the identification of sexually dimorphic genes has been largely overlooked [72]. In the present study, several transcripts related to reproduction were regulated differentially in the males and females. One example is the *calr* gene, which encodes a protein synthetized in the endoplasmic reticulum, which plays a key role in the development of the oocytes and the fertility of the female [73].

Another prominent gene involved in reproduction is *zp3*, which was over-expressed in the liver of females. This gene is translated into a protein which coats the oocyte, acting as the primary receptor for the spermatozoon during fertilization [74]. An interesting feature of the evolution of the teleost fish is that some species synthesize the proteins of the zona pellucida in both the liver and the ovary [75]. Given this, the expression of *zp3* in our transcriptome indicates that this gene is also expressed in the liver of the arapaima.

### Gene expression in the skin of the male and female *A*. *gigas*

In addition to forming the principal physical barrier of the organism to potential environmental damage, the skin also synthesizes mucus, pigments and hormones, and thus plays an important role in the sexual attraction of fish during the breeding season [57]. One other feature of fish skin is the diversity of coloration produced by the presence of pigmented cells, known as chromatophores: yellow xanthophores, red erythrophores, iridescent iridophores, white leucophores and blue cyanophores [76,77], which have a fundamental in the survival and reproduction of many species. Rather than transferring their pigments to other cell types, chromatophores retain their pigments intracellularly. In addition to this natural variation in pigmentation, another unique feature of fish is the temporal variation in skin colors related to nutrition, habitat (e.g., water turbidity), photoperiod, stress, and the spawning period [78]. In *A*. *gigas*, the skin is usually brown to gray or green in color, with occasional red pigments, depending on the type of habitat and diet. The transcriptome analysis revealed the differential expression of genes in the skin of juvenile male and female arapaima, which has the potential for the development of reliable sexing techniques for this commercially-important Amazonian fish. A number of studies have identified sex-specific markers in the transcriptome of fish species, such as the sturgeon, *Acipenser fulvescens* [79], tilapia, *Oreochromis niloticus* [80], Asian arowana, *S*. *formosus* [19], and mosquito fish, *Gambusia affinis* [81].

One of the most prominent genes that was expressed differentially in the skin of the male and female arapaima was sperm acrosome membrane associated protein 4 (*spaca4*), also known as *samp14*. This gene is located on chromosome 19 in humans, and its expression was first reported in the testicles. The final product of *spaca4* is retained in the acrosome membrane of the spermatozoa, and appears to play a role in fertilization in mammals [82]. Lu et al. [83] reported over-expression of this gene in the brains of male catfish (*Pelteobagrus fulvidraco*), indicating that the differential expression of *spaca4* in the two sexes may provide an informative tool for sexing juvenile arapaima. The *spaca4* gene was also found to be up-regulated in analyses of the transcriptomes of two fish, *Trichomycterus areolatus* [84], based on whole-body samples, and *Ictalurus punctatus* [85], focusing on the testicles. While this gene is up-regulated in the males of many different organisms, it was also expressed in the liver libraries of both the male and female arapaima, which indicates that the lack of contigs in the skin of the females does not necessarily mean that the gene was not expressed in this tissue, but rather that there may have been a reduced level of recuperation of the reads from this library.

One other gene expressed intensely in the skin of the males was *mal*, which is usually expressed in the Schwann cells and oligodendrocytes of mammals, being involved in the maintenance of the myelin sheath in the central and peripheral nervous systems [86]. In fish, this gene is known to be over-expressed in juvenile Atlantic salmon (*Salmo salar*) exposed to nutrient-enriched environments [87]. It is important to note that the contigs of both *spaca4* and *mal* were absent from the female libraries.

The expression profiles of the principal genes involved in the pigmentation of the skin of the arapaima were not differentiated between males and females, probably because we analyzed juvenile specimens, which are reproductively immature, or that the number of mapped reads in the transcripts was low. Even so, a number of the other genes included in the present transcriptome analysis can be used to evaluate gene expression the skin of adult arapaima. These genes include the *pmel* which encodes the premelanosome protein, involved in the biogenesis of melanosomes [88,89]. The *matp* (also known as *aim1* or *slc45a2*) is also involved in the first step of melanin synthesis, together with *slc24a5* (which was absent from the arapaima transcriptoma) [90]. In addition to the genes involved in pigmentation, the MSH hormone receptor is also important here, because it is one of the principal regulators of pigmentation in the teleosts, being synthetized in the pituitary gland [78]. It is important to note that most of the pigmentation-related genes identified in this transcriptome are involved in the synthesis, transportation, and storage of melanin, which is responsible for brown and black coloring, and is not normally involved in the differentiation of the coloration between male and female fish (yellow and red). Sexual dimorphism related to the presence/absence of melanin is more common in birds and mammals, which normally have only one type of pigmented cell, the melanocyte, which can produce eumelanin (black/brown) or pheomelanin (yellow/red), and can shift rapidly between the synthesis of these two types of pigment [91].

In fish and other poikilothermic organisms, the pigments in the yellow and red bands are produced primarily by xanthophores (cells with yellow/orange pigments) and erythrophores (cells with red pigments), which may also be produced by a variety of pteridines and carotenoids. Vertebrates can produce pteridine de novo, but cannot synthesize carotenoid pigments in the same way that they produce melanin and other pigments, and normally obtain them through the diet [92]. Given this, the low frequency of the gene involved in the synthesis of carotenoids in the transcriptome of *A*. *gigas* may also reflect the lack of pigment in the fish’s food, given that the captive diet is different from that consumed by wild populations. One other possible explanation is the fact that the specimens analyzed were all juveniles, and still not in breeding condition, given that all previous studies of changes in arapaima pigmentation took place during the rainy season, when spawning begins. The change in the coloration of the male probably serves to attract the female, as observed in other fish species [93,94]. In the Siamese fighting fish, *Betta splendens*, Etan et al. [92] showed that the red coloration is determined by the carotenoids present in the diet, and that this coloration influences the selection of mates by the female.

The transcription factors *sox10*, *mitf*, and *pax3* act synergistically in the regulation of pigmentation and were identified in the genome of *S*. *formosus*, and, in addition to participate in the development of the melanocytes, the *sox10* and *pax3* genes are also involved in the development of the xanthophores [95]. Both *sox10* and *mitf* are involved in Waardenburg’s syndrome, which is characterized by the lack of pigmentation of the hair, eyes, and skin, and is normally associated with the deletion of *sox10* [96]. The *csf1r* gene expressed only on the skin of *A*. *gigas* was also identified in the genome of *S*. *formosus* associated in the development of the xanthophores, as well as the *xdh* and *mycbp2* was associated in the development of the pteridines [95]. These genes should be investigated further to examine their putative role in the regulation of pigmentation in the arapaima.

The analysis of the Vitellogenin (*vtg*) is one of the few techniques available for the identification of the sex of adult arapaima, given that it is abundant in the plasma of the adult females, but is usually absent in that of the males and juveniles [22]. In the present study, however, this gene, which responsible for the synthesis of the precursor of yolk protein, was expressed in the skin of both sexes. While the identification of *vtg* in the blood is the principal technique used to sex adult arapaima [23], then, the expression of *vtg* gene in the skin of both sexes indicates that this technique may not be reliable for juvenile arapaima. One unexpected finding was the absence of vitellogenin from the liver of the arapaima, in particular in the females. This is probably because the specimens examined in the present study were juveniles and the protein was either absent from their tissue or found in prohibitively low concentrations.

## Conclusion

This is the first transcriptome published for the Amazonian fish *A*. *gigas* using RNA-seq. The distribution of the transcripts in distinct functional categories based on the Gene Ontology terminology highlighted the effectiveness of pyrosequencing in the 454 Roche platform and *de novo* assembly in a non-model species. The findings of the present study provided the first evidence of sexually dimorphic gene expression in the juvenile stage of the arapaima, based on comparisons of the expression profiles of the liver and skin tissue. The differential expression of genes in the skin of immature male and female arapaima can be used to develop reliable sexing techniques to support the development of effective captive rearing protocols for this ecologically vulnerable, but economically valuable Amazonian species. In addition, to the genes with differential expression a number of genes and proteins involved in a variety of biological processes were identified in the skin and liver tissue, and should be investigated further. This informative transcriptome provides valuable new data to increase genomic resources of *Arapaima gigas*.

## Supporting information

S1 Fig Distribution of contigs length obtained by pyrosequencing in the platform 454 Roche. (TIF)

S2 Fig Gene ontology (GO) at the second level of GO terms obtained for all transcripts of the male, all transcripts of the female and the complete transcriptome of *A*. *gigas*. (TIF)

S3 Fig Gene ontology (GO) at the second level of GO terms obtained for skin and liver transcripts of *A*. *gigas*. (TIF)

S1 Table Full list of gene with acession number, mappead reads counts, RPKM, Foldchange, and log2 Fold Change, obtained for the liver and skin libraries of *A*.*gigas*. (CSV)

S2 Table Up-regulated genes between male and female liver of *A*. *gigas*. (CSV)

S3 Table Up-regulated genes between male and female skin of *A*. *gigas*. (CSV)

S4 Table List of the genes used to generate the heatmap, with their respective RPKMs. (CSV)

S5 Table List of the genes cited in the present manuscript. (CSV)
