# Supplementary figures and images for: De novo transcriptome based on next-generation sequencing reveals candidate genes with sex-specific expression in Arapaima gigas (Schinz, 1822), an ancient Amazonian freshwater fish
Source: PLoS One. 2018 Oct 29;13(10):e0206379. doi: 10.1371/journal.pone.0206379 (PMC6205615; doi:10.1371/journal.pone.0206379)

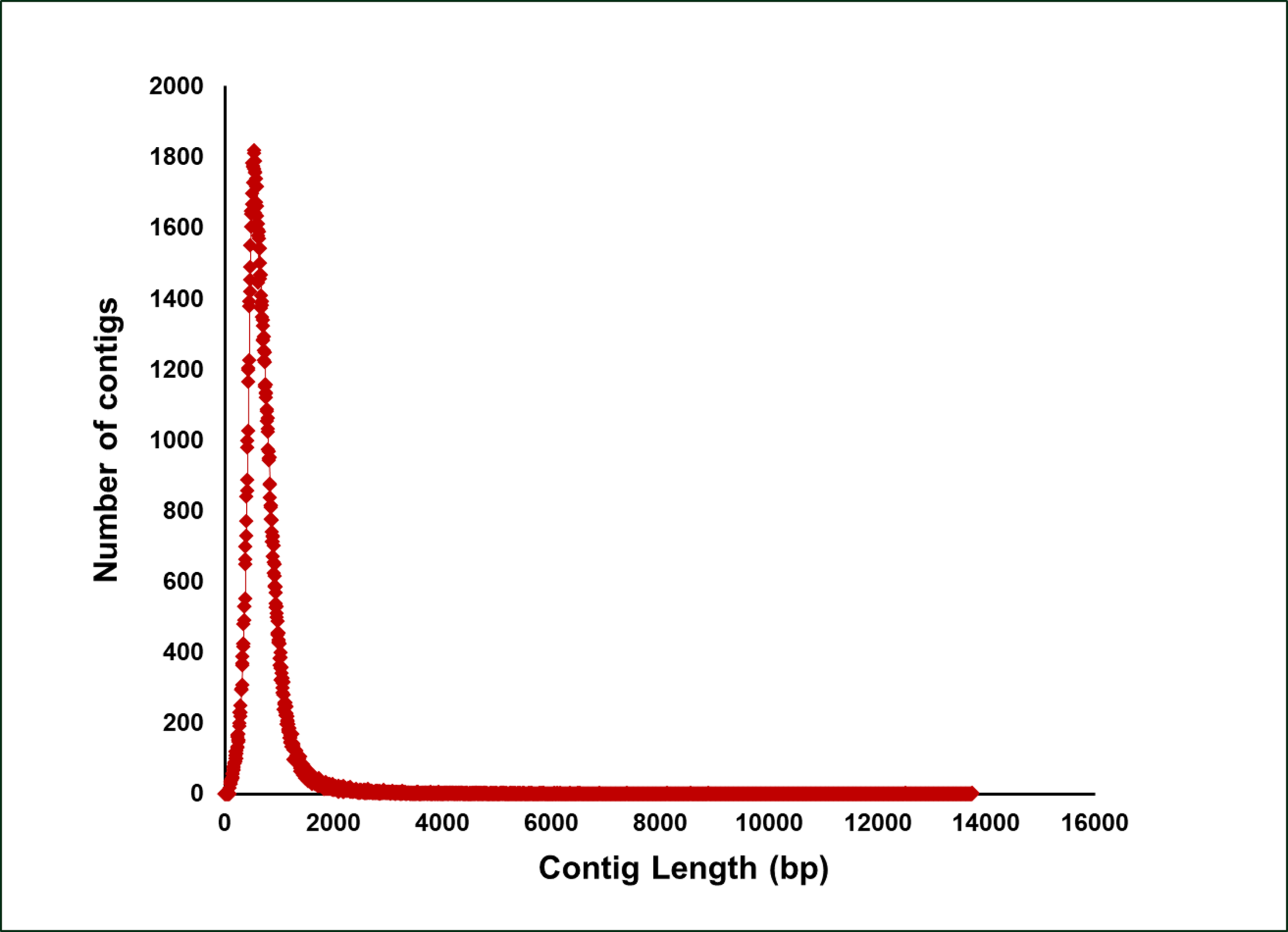

Supplement: S1 Fig — (TIF) [file pone.0206379.s001.tif]

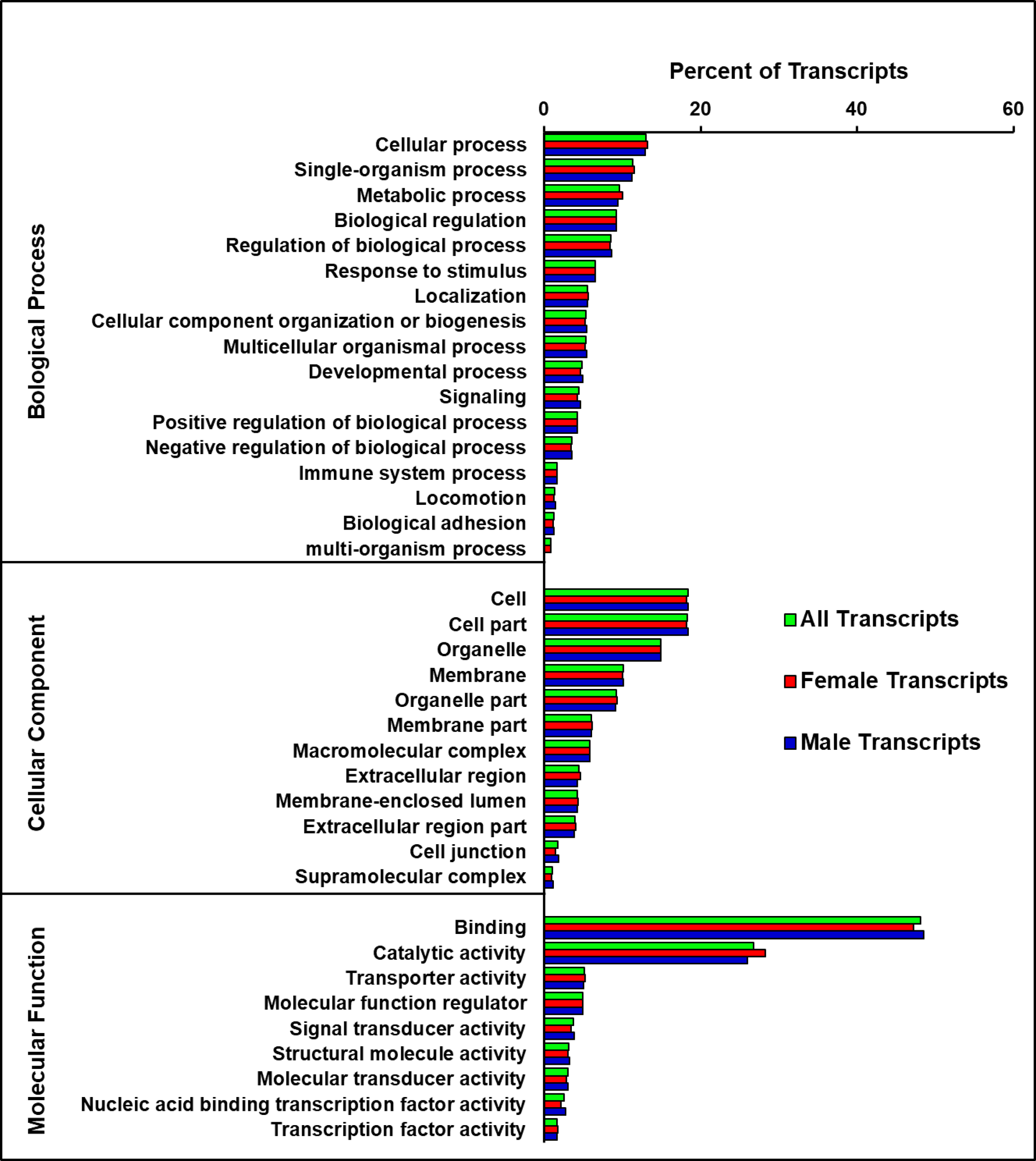

Supplement: S2 Fig — (TIF) [file pone.0206379.s002.tif]

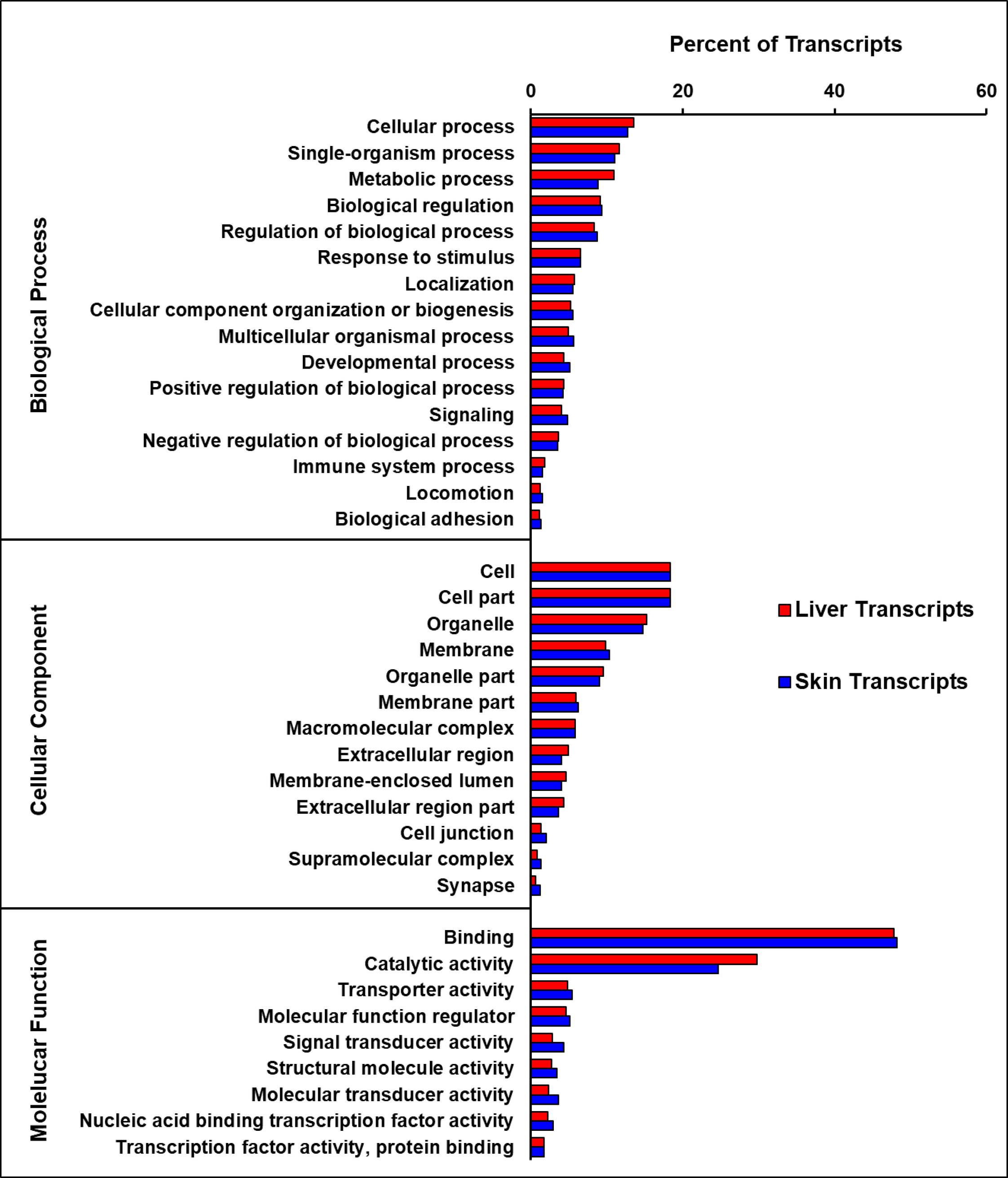

Supplement: S3 Fig — (TIF) [file pone.0206379.s003.tif]
